# Electrochemical Water Oxidation and CO_2_ Reduction with a Nickel Molecular Catalyst

**DOI:** 10.3390/molecules29030578

**Published:** 2024-01-24

**Authors:** Hengxin Jian, Mengyu Lu, Haowen Zheng, Shengrui Yan, Mei Wang

**Affiliations:** School of Materials Science and Engineering, Institute for New Energy Materials & Low Carbon Technologies, Tianjin University of Technology, Tianjin 300384, China; jianhx0306@163.com (H.J.); lumengyu@stud.tjut.edu.cn (M.L.); zhenghaowen@stud.tjut.edu.cn (H.Z.); ysr2109746471@stud.tjut.edu.cn (S.Y.)

**Keywords:** transition metal complex, water oxidation, carbon dioxide reduction, electrocatalysis, synergistic catalysis

## Abstract

Mimicking the photosynthesis of green plants to combine water oxidation with CO_2_ reduction is of great significance for solving energy and environmental crises. In this context, a trinuclear nickel complex, [Ni^II^_3_(paoH)_6_(PhPO_3_)_2_]·2ClO_4_ (**1**), with a novel structure has been constructed with PhPO_3_^2−^ (phenylphosphonate) and paoH (2-pyridine formaldehyde oxime) ligands and possesses a reflection symmetry with a mirror plane revealed by single-crystal X-ray diffraction. Bulk electrocatalysis demonstrates that complex **1** can homogeneously catalyze water oxidation and CO_2_ reduction simultaneously. It can catalyze water oxidation at a near-neutral condition of pH = 7.45 with a high TOF of 12.2 s^−1^, and the Faraday efficiency is as high as 95%. Meanwhile, it also exhibits high electrocatalytic activity for CO_2_ reduction towards CO with a TOF of 7.84 s^−1^ in DMF solution. The excellent electrocatalytic performance of the water oxidation and CO_2_ reduction of complex **1** could be attributed to the two unique *µ*_3_-PhPO_3_^2−^ bridges as the crucial factor for stabilizing the trinuclear molecule as well as the proton transformation during the catalytic process, while the oxime groups modulate the electronic structure of the metal centers via π back-bonding. Therefore, apart from the cooperation effect of the three Ni centers for catalysis, simultaneously, the two kinds of ligands in complex **1** can also synergistically coordinate the central metal, thereby significantly promoting its catalytic performance. Complex **1** represents the first nickel molecular electrocatalyst for both water oxidation and CO_2_ reduction. The findings in this work open an avenue for designing efficient molecular electrocatalysts with peculiar ligands.

## 1. Introduction

Converting carbon dioxide into high-value-added fuel and chemicals has been considered to be a critical route to achieve carbon neutrality and mitigate energy issues around the world [1,2,3,4,5,6,7]. Among various electrocatalytic carbon dioxide reduction products, CO serves as a crucial building block for the production of numerous important chemicals and materials. In addition, CO can be combined with hydrogen to produce syngas, which is a versatile feedstock for the production of fuels [8,9]. The electrocatalytic reduction of CO_2_ to CO only requires a transfer process of two electrons, which is the main product of the homogeneous electrocatalytic reduction of carbon dioxide. In the photosynthesis of green plants, carbon dioxide is reduced, while water is oxidized to release oxygen. During this process, the half reaction of water oxidation (OER) could provide enough protons and electrons for the CO_2_ reduction reaction [10,11,12,13,14,15]. In addition, the OER is also the half reaction of water-splitting for hydrogen evolution, which is a crucial process in the field of clean energy conversion and storage [16]. Moreover, oxygen is an essential component in many industrial fields, including combustion, chemical synthesis, and wastewater treatment. The preparation of oxygen often requires separation from liquid air. The OER process can provide a sustainable and efficient method for producing oxygen on demand, ensuring a stable supply for these industries [17]. Therefore, mimicking the photosynthesis of green plants to combine water oxidation with CO_2_ reduction in one system is of great significance for solving energy and environmental crises [18,19,20,21,22,23,24]. Meanwhile, the utilization of surplus renewable electricity in electrocatalytic water oxidation and carbon dioxide reduction generated from hydro, wind, and solar power, among others, has become a current hot research topic [25]. Nevertheless, the two reactions of water oxidation and CO_2_ reduction involve multi-electron and -proton transfer, resulting in extremely high thermodynamic barriers and very slow reaction kinetics [26,27,28,29,30,31]. In this regard, enormous efforts are required to develop efficient and robust catalysts with low overpotential and high Faraday efficiency to overcome the above obstacles. Molecular catalysts can be easily synthesized and modified, allowing for the development of tailored catalysts with improved performance. By tuning the ligand environment and controlling the coordination geometry of the center of the molecular catalysts, researchers can flexibly optimize the catalyst’s parameters, such as overpotential, selectivity, and Faradaic efficiency. Hence, molecular catalysts have shown to be a promising alternative in the field of water oxidation and CO_2_ reduction [32].

Transition-metal-based complexes, such as Mn, Fe, Ru, Co, and Cu, have been investigated intensively as homogeneous catalysts for water oxidation. By contrast, nickel complexes for the exploration of water oxidation are relatively rare [33,34]. Lu’s group developed a mononuclear nickel complex with a macrocyclic ligand that can homogeneously catalyze water oxidation under neutral conditions with a low overpotential of 170 mV and an FE of 97.5%, exhibiting excellent reactivity and stability [35]. Sun’s team reported a highly efficient and robust water oxidation electrocatalyst, Ni^II^-PY5 [PY5 = 2,6-bis(1,1-bis(2-pyridyl)ethyl)pyridine)], manifesting a high rate constant of 1820 M^−1^s^−1^ and a high FE over 90% under the alkaline conditions of pH 10.8 [36]. In 2020, our group successfully characterized two biologically inspired tetranuclear nickel(II) catalysts, [Ni^II^_4_(L-H)_4_(CH_3_COO)_3_]·Cl (**1**) and [Ni^II^_4_(L-H)_4_(CH_3_COO)_4_]·2CH_3_OH (**2**) (L = di(pyridin-2-yl)methanediol). The two water-soluble complexes can efficiently electrocatalyze water oxidation at relatively low overpotentials (η) with high TOFs of about 139 s^−1^ (**1**) and 69 s^−1^ (**2**) at pH 12.32, respectively [37]. Very recently, Zhang et al. successfully obtained a trinuclear Ni homogeneous catalyst for water oxidation, displaying outstanding stability under neutral conditions with a high FE ≈ 93% [38].

On the other hand, the well-designed nickel complexes also have great potential to serve as efficient molecular electrocatalysts for CO_2_ reduction reactions (CO_2_RRs). Hollis et al. constructed a Ni(II) complex coordinated with a N-heterocyclic carbene pincer ligand, which can reduce CO_2_ to CO and formate, with FE values of 34% and 47%, respectively [39]. Rosenthal et al. reported four newly designed substituted cyclen nickel mononuclear complexes that demonstrated great stability and selectivity for the conversion of CO_2_ to CO, with the FE values reaching up to 80% [40]. Recently, Han, Eisenberg, and co-workers unprecedentedly discovered that nickel thiolate complexes could electrocatalyze a CO_2_ reduction to C_3_ hydrocarbons with a molecular electrocatalyst for the first time [41]. Nevertheless, it is worth noting that there have been rare reports on the simultaneous application of nickel molecules in electrocatalytic water oxidation and CO_2_ reduction.

Phosphonate ligands can adopt different coordination modes, such as bidentate, tridentate, or tetradentate, enabling the formation of diverse and well-defined coordination geometries. This flexibility allows for the design and synthesis of complexes with specific structural and electronic properties, making phosphonate ligands useful in various areas such as catalysis [42,43], materials science [44,45,46,47,48], and bioinorganic chemistry [49]. Moreover, phosphonate ligands often possess functional groups that can be easily modified or derived, providing opportunities for the further tuning of their properties, such as enhanced redox activity and selectivity in catalysis. Our group constructed two trinuclear copper phosphonate complexes, [Cu^II^_3_(pda)_3_(tBuPO_3_)] and [Cu^II^_3_(pda)_3_(PhPO_3_)] (pdaH_2_ = 2, 6-pyridinedicarboxylic acid, ^t^Bu/PhPO_3_^2−^ = tertbutyl/pheny-phosphonate), and found that the two different phosphonate ligands have diverse effects on electrocatalytic water oxidation with distinct TOF values [50]. Llobet isolated a Ru(III) complex, [Ru^III^(^t^PaO-κ-N^2^OPOC)(py)_2_]^2−^, with the 3-(hydroxo-[2,2′:6′,2″-terpyridine]-6,6″-diyl)bis(phosphonate) ligand (^t^PaO^5−^), which displays excellent electrocatalytic performance towards water oxidation owing to the phosphonate group reducing the activation energy of the key reaction step [51]. Therefore, the modification of phosphonate ligands can lead to the development of electrocatalysts that meet the requirements of catalytic applications.

Herein, we applied the phenylphosphonate ligand combined with a 2-pyridyl oxime group and nickel metal to build a trinuclear Ni(II) complex, [Ni^II^_3_(paoH)_6_(PhPO_3_)_2_]·2ClO_4_ (**1**). Our systematic electrocatalysis exploration shows that complex **1** can act as a bifunctional catalyst for water oxidation and CO_2_ reduction with great activity and stability, which could be attributed to the synergistic catalytic effect between the three nickel metals and the ligands. The two *µ*_3_-PhPO_3_^2−^ ligands can bind three Ni(II) ions from the top and bottom simultaneously, greatly enhancing the stability of the cluster. Meanwhile, the two unique *µ*_3_-PhPO_3_^2−^ bridges are the crucial factor for proton transformation during the catalytic process. In addition, N atoms on the oxime group could serve as an edge base, which can donate σ electrons to the Ni metal centers while obtaining π electrons through π back-bonding to modulate the electronic structure of the metal centers [52,53,54,55,56,57,58,59,60,61,62]. The two kinds of ligands could highly boost the electrocatalytic performance of complex **1**. Complex **1** represents the first nickel molecular electrocatalyst for both water oxidation and CO_2_ reduction.

## 2. Results and Discussion

### 2.1. Description of Crystal Structures

**Figure 1:**
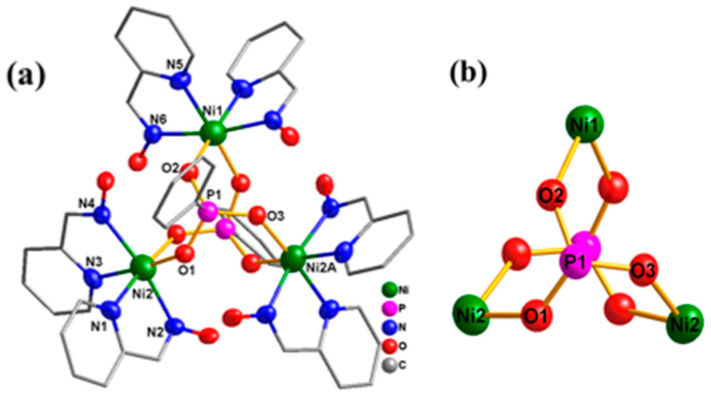
(**a**) Structure diagram of trinuclear cation of complex **1**; the hydrogen atoms and anions have been omitted for clarity. (**b**) The inorganic core of complex **1**.

X-ray single-crystal diffraction analysis indicates that complex **1** belongs to the monoclinic space group P2/n with a reflection symmetry (Table 1). The cationic portion of complex **1** is shown in Figure 1, and the selected bond lengths and bond angles are displayed in Table S1. Complex **1** has a plane symmetry with Ni1 in the mirror plane. According to the bond valence sum calculations, the nickel atoms of complex **1** are all in the +2 oxidation state [63]. The periphery around each Ni^II^ ion is coordinated by two N,N′-chelated paoH ligands, and the three Ni^II^ ions are bridged together with two η^1^: η^1^: η^1^: *μ*_3-_PhPO_3_^2−^ from the top and bottom simultaneously in a triangular configuration. Meanwhile, the oxime groups’ =N–OH could link with the O atoms of the PhPO_3_^2−^ to form strong intramolecular hydrogen bonds, which are conducive to maintaining the stability of trinuclear units. As presented in Figure S1, the infrared spectrum (IR) of complex **1** shows absorption peaks between 3300 and 3000 cm^−1^, which correspond to the stretching vibration of C-H on the unsaturated carbon of the benzene ring. The absorption peak at 1095 cm^−1^ is due to the stretching vibration of the P=O double bond in the phenylphosphonate ligand. The absorption peaks between 1630 and 1430 cm^−1^ can be attributed to the stretching vibration of C=C and C=N in the pyridine ring, confirming the coordination of the paoH ligand. In addition, we have conducted X-ray photoelectron spectroscopy (XPS) of complex **1** (Figure S2), which further confirms the presence of the elements Ni, O, C, P, N, and Cl in the sample [64]. As presented in Figure S2a, the peaks at 531.8 eV can be assigned to the bind energy of O^2−^ (1*s*). As shown in Figure S2b, the two significant peaks with the binding energies of 872.3 eV and 855.2 eV can be attributed to Ni^2+^ (2*p*_1/2_) and Ni^2+^ (2*p*_3/2_), respectively, which affirms the chemical state of Ni is +2. Meanwhile, the other two peaks at 879.4 eV and 860.5 eV are assigned to satellite peaks.

### 2.2. Electrochemistry under Argon Atmosphere

**Figure 2:**
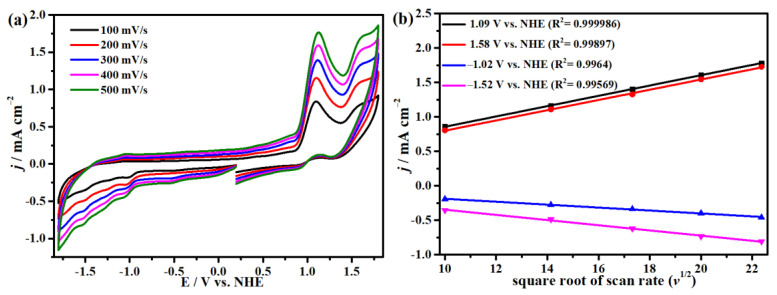
(**a**) Cyclic voltammogram of complex **1** in DMF solution including 0.1 M ^n^Bu_4_NPF_6_ at scan rates ranging from 100 to 500 mV s^−1^. (**b**) The graph shows the linear relationships between the irreversible peak currents of complex **1** and the square root of the scanning rates at potentials of −1.52, −1.02, 1.09, and 1.58 V.

Figure 2a shows the cyclic voltammogram (CV) curves of complex **1**, obtained at different scanning rates (100–500 mV s^−1^) in 0.1 M ^n^Bu_4_NPF_6_/DMF solution under argon (Ar) atmosphere (all potentials are versus the NHE electrode), which displays the redox performance of compound **1**. In addition, as illustrated in Figure 2b, the cathode (at −1.02, −1.52 V) and counter electrode (at 1.09, 1.58 V) current peaks at different scanning rates have good linear correlations with the square root of the scanning rates, which proves that all the electrode processes are primarily diffusion controlled.

### 2.3. Electrocatalytic Water Oxidation

**Figure 3:**
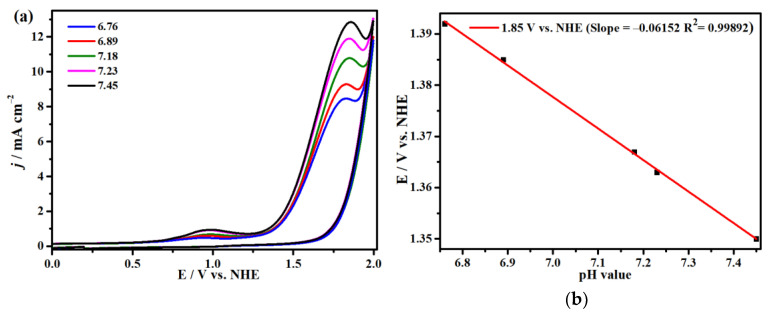
(**a**) Cyclic voltammograms of complex **1** are carried out at different pH levels using a glassy carbon electrode as a working electrode. (**b**) The image displays the linear relationships between initial potentials of the complex **1** and pH values at 1.85 V (scan rate = 100 mV s^−1^).

**Figure 4:**
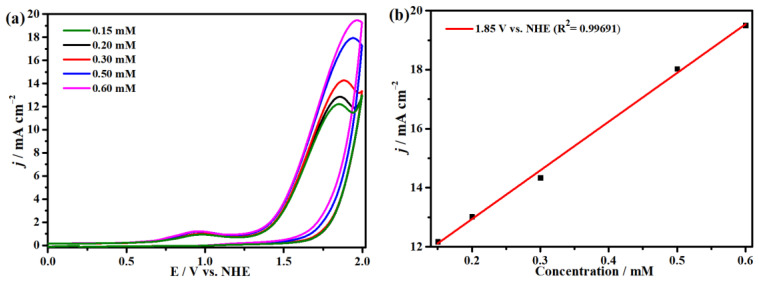
(**a**) Cyclic voltammograms at different concentrations of complex 1 in 0.1 M sodium acetate buffer solution at a scanning rate of 100 mV s^−1^. (**b**) The linear relationships between different concentrations of complex **1** and the peak currents at 1.85 V and the optimal pH of 7.45.

Firstly, to investigate the effect of different pH values on electrocatalytic water oxidation, we discussed the CVs of complex **1** in acidic and alkaline circumstances in 0.1 M sodium acetate (NaOAc) buffer solution adjusted by acetic acid and sodium hydroxide. As presented in Figure 3a, complex **1** displays the largest oxidation current and an enhanced irreversible oxidation peak under the condition of pH = 7.45 at the potential of 1.85 V. Figure 3b reveals the linear relationship between different pH values with a slope of 54 mV pH^−1^ and different initial potentials at 1.85 V, which demonstrates that catalytic water oxidation should be a PCET (proton-coupled electron transfer) process relating 1H^+^/1e^−1^ [65]. After continuing to add 0.1 M NaOH solution, it was found that the oxidation currents gradually decreased with the increase in pH values, which further verified that complex **1** has the best catalytic effect for water oxidation at pH = 7.45, as displayed in Figure S5. Moreover, no oxidation peak was discovered in the aqueous solution of NaOAc without complex **1**, indicating that it is complex **1** that is responsible for the catalytic water oxidation in the system (Figures S3 and S6). Additionally, at 1.85 V, a lot of bubbles can be noticed on the surface of the GC electrode, and the oxygen obtained can be detected with an Ocean Optics NeoFox–GT oxygen sensor. The above outcomes verify that compound **1** can catalyze water oxidation at 1.85 V. Furthermore, we have also explored the relationship between the peak currents and concentrations of complex **1** (Figure 4). It is found that the catalytic current peaks increase linearly with the concentrations of complex **1**, disclosing that the catalytic water oxidation reaction at 1.85 V is a first-order process (Figure 4b) [66].

Based on the half-peak potential of the CVs (*E_real_*(O_2_/H_2_O)) and the overpotential (*η*) defined in Formulas (1) and (2), the overpotential of complex **1** for electrocatalytic water oxidation is about 560 mV at pH = 7.45, which is comparable to those of other nickel complexes reported previously (Table S2).

E*_theory_* (O_2_/H_2_O) = 1.23 − 0.059pH (1)

*η* = E*_real_* (O_2_/H_2_O) − E*_theory_* (O_2_/H_2_O) (2)

**Figure 5:**
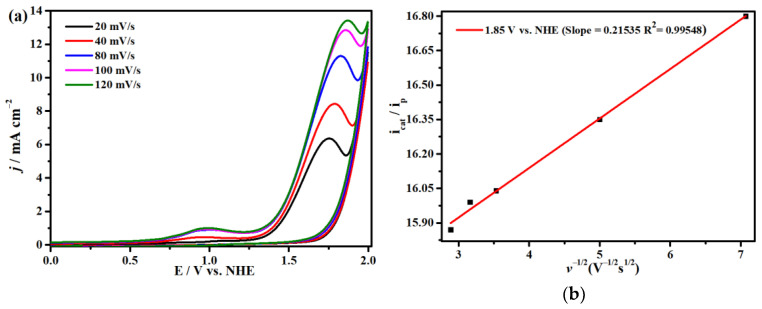
(**a**) Cyclic voltammograms of 0.2 mM complex **1** under 1 atm Ar at scan rates ranging from 20 to 120 mV s^−1^. (**b**) Linear relationship between the value of *i_cat_*/*i_p_* and the inverse function of the square root of scanning rate at 1.85 V (0.1 M sodium acetate buffer solution).

As shown in Figure 5, in order to further explore the kinetic information of complex **1** for water oxidation, the cyclic voltammogram plots at different scan rates were carried out at the best condition of pH = 7.45. It is revealed that *i_cat_/i_p_* has a linear relationship with the inverse function of the square root of the rate (*v*^−1/2^). The pseudo-first-order rate constant *k_cat_* can be ascertained using Formula (3), while *k_cat_* is always regarded as the turnover frequency (TOF) of the electrocatalytic water reaction. On the basis of Equation (3), the calculated TOF value is 12.2 s^−1^ at 1.85 V, which is higher than the other reported nickel molecular catalysts for water oxidation (Table S2) [67,68,69,70,71,72,73].

(3) $$\frac{i_{cat}}{i_{p}}\;=0.356n_{cat}\sqrt{\frac{k_{cat}}{v}}$$

where *i_cat_* is the catalytic current, *i_p_* is the peak current measured without substrates, *v* is the scanning rate (0.1 V s^−1^), and *n_cat_* is the number of electrons involved in the catalytic reaction (*n_cat_* = 4).

**Figure 6:**
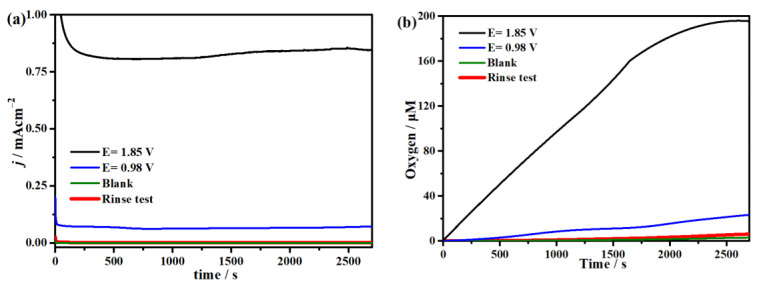
(**a**) CPE with and without complex **1** at a potential of 0.98 V (blue line) and 1.85 V (black line) on the FTO electrode. (**b**) Dissolved oxygen curves with and without complex **1** at pH = 7.45 during electrolysis.

Moreover, a controlled potential electrolysis (CPE) experiment employing fluorine-doped tin oxide (FTO) conductive glass with a surface area of 1.0 cm^2^ as the working electrode was performed to investigate the water oxidation activity of complex **1** in 0.1 M NaOAc at pH = 7.45 at 0.98 V and 1.85 V. At the same time, the oxygen density was recorded with the oxygen sensor in the solution in situ so as to confirm the generated oxygen content. (The oxygen density in the top space of the electrolytic cell is lower than the detection limit of the sensor.) As depicted in Figure 6, in the blank solution without complex **1**, the oxygen content and observed current density are near zero during the 2700 s of electrolysis at applied potentials of 0.98 V and 1.85 V (red line), showing that no catalysis occurred. However, after 0.2 mM complex **1** was added under the same conditions, the electrolytic current density and oxygen content increased sharply to reach as high as ~0.8 mA cm^−2^ and 195 µM, respectively, at 1.85 V (Figure 6a,b, black line). Meanwhile, the current density and oxygen content can only obtain values of 0.07 mA cm^−2^ and 23 µM at 0.98 V (blue line). Based on the results of the CPE, the calculated Faraday efficiency of complex **1** at 1.85 V is ~95%, which is higher than those of other reported nickel catalysts (Table S2). The electrolytic current density of complex **1** remains stable during the 2700 s, illustrating that catalyst **1** has a great stability. As displayed in Figure S7b, the FTO electrodes before and after electrocatalysis were examined by scanning electron microscopy (SEM), and no metallic oxides were observed on the surface of the electrolyzed FTO. More importantly, as shown in Figure S8a, the continuous cyclic voltammetry curves for water oxidation are repeated well after ten cycles, which further verifies the high stability of complex **1**. Furthermore, the rinse test conducted on the FTO glass electrode after electrocatalysis displays barely any current density, which is similar to the blank test before catalysis, demonstrating no nanomaterials were deposited on the working electrode during catalysis (Figure 6, red line). What is more, Figure S9a exhibits the in situ ultraviolet–visible (UV-vis) spectroelectrochemistry of complex **1** during the 4000 s CPE, which reveals there is almost no difference in the spectra (the schematic diagram of the in situ UV-vis spectral electrochemistry is shown in Figure S10). All the above results can prove that complex **1** can efficiently catalyze water oxidation with high stability, which could be because the two *µ_3_*-PhPO_3_^2−^ ligands can bind three Ni(II) ions from the top and bottom simultaneously, greatly enhancing the stability of the cluster during catalysis; most importantly, the two unique *µ_3_*-PhPO_3_^2−^ bridges act as the crucial factor for the proton transformation during the catalytic process. Additionally, N atoms on the oxime group could serve as an edge base, which can donate σ electrons to the Ni metal centers while obtaining π electrons through π back-bonding to modulate the electronic structure of the metal centers for promoting the catalytic performance of complex **1** [74,75]. Therefore, apart from the cooperation effect of the three Ni centers for catalytic water oxidation, simultaneously, the two kinds of ligands in complex **1** can also synergistically coordinate the central metal, thereby greatly enhancing its catalytic performance.

### 2.4. Electrocatalytic CO_2_ Reduction

**Figure 7:**
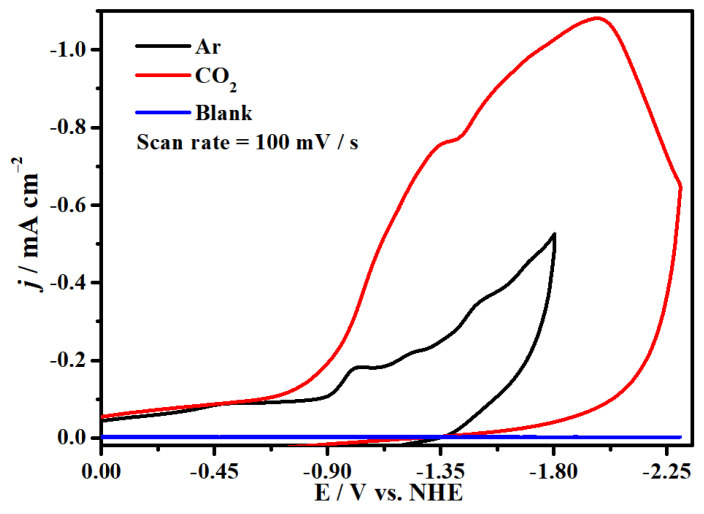
Cyclic voltammograms of 0.2 mM complex **1** under 1 atm Ar (black line) and CO_2_ (red line) in DMF containing 0.1 M ^n^Bu_4_NPF_6_ solution and cyclic voltammogram of DMF/0.1 M ^n^Bu_4_NPF_6_ solution only (blue line).

**Figure 8:**
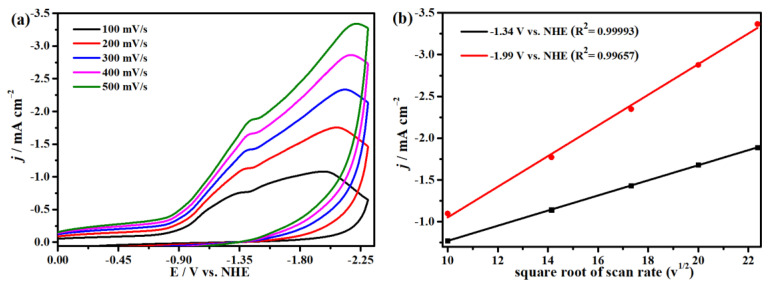
(**a**) Cyclic voltammograms of 0.2 mM complex **1** at different scanning rates from 100 to 500 mV s^−1^ under CO_2_-saturated DMF containing 0.1 M ^n^Bu_4_NPF_6_ solution. (**b**) The linear relationships between the square root of complex **1** at different sweep rates and the peak currents are shown at −1.34 and −1.99 V.

**Figure 9:**
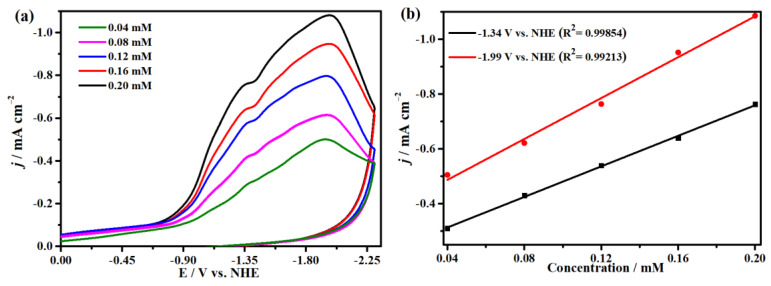
(**a**) Cyclic voltammograms of complex **1** with different concentrations at 100 mV s^−1^ with glass carbon electrode. (**b**) The linear relationships between the concentrations of complex **1** and the peak currents.

**Figure 10:**
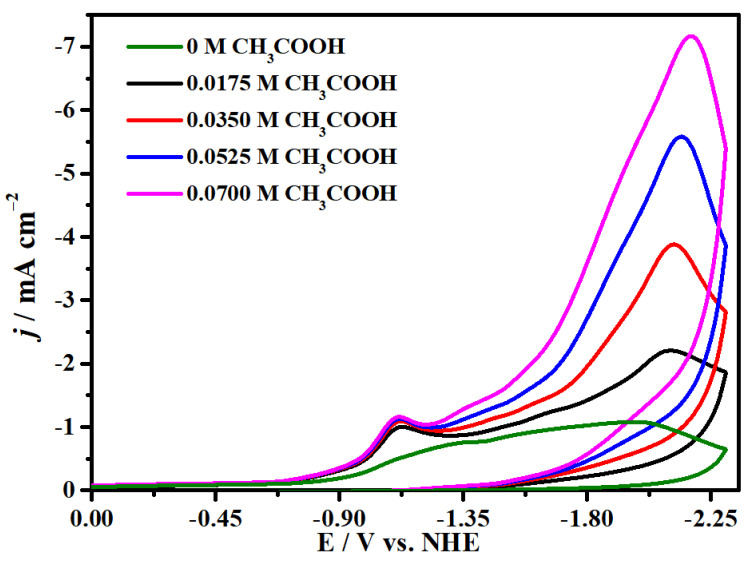
Cyclic voltammograms of different concentrations of acetic acid solution added to complex **1**.

The electrocatalytic properties of complex **1** for CO_2_ reduction in DMF solution with 0.1 M ^n^Bu_4_NPF_6_ as supporting electrolyte were investigated with a glassy carbon (GC) electrode as the working electrode in the saturated CO_2_ atmosphere. We firstly compared the cyclic voltammograms of complex **1** in Ar and saturated CO_2_ at 100 mV s^−1^ and found two enhanced, irreversible reduction waves at −1.34 and −1.99 V (versus NHE), suggesting electrocatalytic CO_2_ reduction occurred at the two potentials (Figure 7 and Figure S4). Meanwhile, it has good repeatability at the different scanning rates without new oxidation or reduction peaks appearing, proving the stability of complex **1** for CO_2_ reduction, as indicated in Figure 8. Moreover, Figure 8b shows that the peak currents have linear relationships with the square roots of the scanning rates, indicating that all processes in the solution are controlled by diffusion. In addition, the concentrations of complex **1** have a linear relationship with the catalytic currents at −1.99 V and −1.34 V, exhibiting that the rate-determining step of the catalytic conversion of CO_2_ to CO is a first-order reaction (Figure 9). In addition, we used the FTO glass electrode with a surface area of 1.0 cm^2^ to carry out the CPE for up to 4000 s to prove the stability of complex **1**, while CO was detected by GC (gas chromatography) analysis (Table S3). Generally, the process of the electrocatalytic reduction of carbon dioxide to carbon monoxide is a proton-coupled electron transfer (PCET) process; thus, adding protons to the system can promote the electrocatalytic process [76]. Accordingly, we also explored the effect of the proton source on the catalytic CO_2_-to-CO process under the same conditions. As demonstrated in Figure 10, the reduction peak current density increases with the addition of acetic acid (CH_3_COOH) at −1.99 V and −1.34 V, which demonstrates that the addition of a proton source can greatly improve the reduction activity of complex **1** for CO_2_ [77,78].

**Figure 11:**
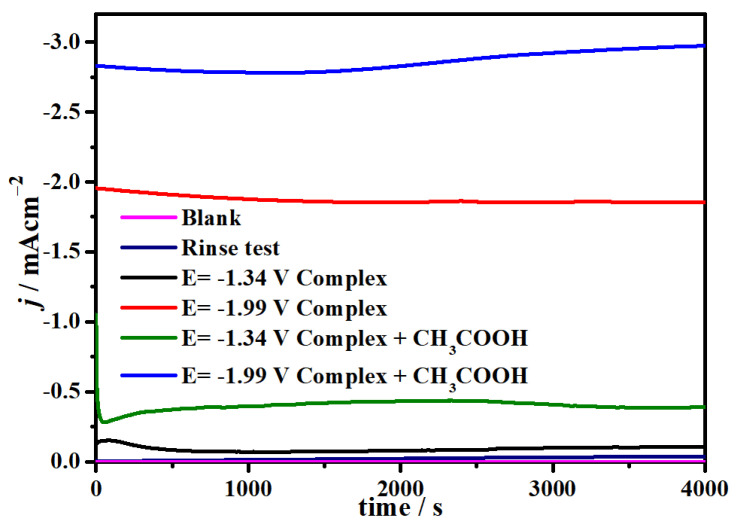
CPE of 0.2 mM complex **1** in DMF/0.1 M ^n^Bu_4_NPF_6_ at −1.34 (black line) and −1.99 V (red line) or with 0.0175 mM CH_3_COOH added at the potentials of −1.34 (green line) and −1.99 V (blue line). CPE of no complex **1** (rose line) under 1 atm Ar on the FTO working electrode and during rinse test (navy blue).

To further investigate the electrocatalytic ability of complex **1** for CO_2_ reduction, a series of CPE experiments was carried out in CO_2_-saturated 0.1 M ^n^Bu_4_NPF_6_/DMF solution and also with added 0.0175 M CH_3_COOH as the proton source. At −1.99 V and −1.34 V, the reduction current density of complex **1** can reach −1.86 (red line) and −0.08 mA cm^−2^ (black line), as indicated in Figure 11. When 0.0175 mM CH_3_COOH was added, it was found that the current density greatly increased at −1.99 V, reaching as high as −2.85 mA cm^−2^ (Figure 11, blue line). In addition, the reduction current was close to zero and no CO was detected in the blank solution without complex **1**, indicating that only complex **1** can catalyze the CO_2_ reduction. Additionally, no formic acid or ethanol was detected in the solution after catalysis. During the CPE of 4000s, the electrolysis current density was stable and no obvious sediment was found on the surface of the FTO conductive glass electrode, which confirms the great stability of complex **1** for the reduction of CO_2_. In addition, to further explore the stability of complex **1** for electrocatalytic CO_2_ reduction, we provided 10 cycles of CVs for complex **1** at the scan rate of 100 mV/s for electrolysis (Figure S8b), which repeated well, indicating that complex **1** is a stable electrocatalyst for CO_2_ reduction.

According to the following Formula (4), the turnover frequency (TOF) for the electrocatalytic CO_2_ of complex **1** can be calculated.

(4) $$\mathrm{TOF}=\frac{Fvn_{p}^{3}}{RT}(\frac{0.4463}{n_{cat}}{)}^{2}(\frac{i_{cat}}{i_{p}}{)}^{2}$$

where *F* is the Faraday constant (96,485 C·mol^−1^), *v* is the scanning rate used (0.1 V s^−1^), *n_p_* is the number of electrons involved in the non-catalytic oxidation–reduction reaction (*n_p_* = 1), *R* is the gas constant (8.314 J·K^−1^·mol^−1^), *T* is the temperature (298.15 K), *n_cat_* = 2 indicates the reduction of CO_2_ to CO, and *i_p_* and *i_cat_* are identified as peak currents under Ar and CO_2_, respectively.

The TOF is separately calculated as 7.84 s^−1^ (*i_cat_/i_p_* = 6.3) in CO_2_-saturated 0.1 M ^n^Bu_4_NPF_6_/DMF solution with 0.0175 M CH_3_COOH at −1.99 V, which is comparable to those reported nickel-based homogeneous catalysts (Table S4) [76,77,78,79,80,81]. However, due to the process being controlled by diffusion, for most molecular catalysts, the product of the electrocatalytic CO_2_ is carbon monoxide, which is very different from other heterogeneous catalysts for producing formic acid and formate with Ni-based anodes [82,83,84,85]. Then, the rinse test was conducted on the FTO glass electrode after electrocatalysis, and no obvious current density was observed, which was similar to the blank solution before catalysis (navy blue line), ensuring that complex **1** is a stable homogeneous catalyst. We also conducted SEM for the FTO electrode and found that no deposits formed on the electrode surface compared with the clean glass electrode (Figure S7a,c). In addition, the in situ UV-vis spectroelectrochemistry of complex **1** was detected during the 4000 s CPE, and there was almost no difference in each 600 s of the spectra (Figures S9b and S10). All results demonstrated the high stability of catalyst **1**.

## 3. Experimental Sections

### 3.1. Chemical Materials and Methods

#### 3.1.1. Chemical Materials

Triethylamine (Et_3_N, 99.5%), pyridine-2-aldoxime (paoH, 98%), phenylphosphonic acid (C_6_H_7_O_3_P, 98%), and acetic acid (CH_3_COOH, 36%) were purchased from Shanghai Aladdin Biochemical Technology Co., Ltd. (Shanghai, China). Nickel(II) perchlorate hexahydrate (Ni(ClO_4_)_2_·6H_2_O, 98%) and tetrabutylammonium hexafluorophosphate (^n^Bu_4_NPF_6_, 98%) were purchased from Shanghai McLean Biochemical Technology Co., Ltd., Shanghai, China. Methyl alcohol (MeOH, 99.5%), sodium acetate anhydrous (CH_3_COONa, 99%), anhydrous acetonitrile (MeCN, 99.8%), N,N-Dimethylformamide (DMF, 99.8%), and sodium hydroxide (NaOH, 9%) were purchased from Sinopharm Chemical Reagent Co., Ltd. (Shanghai, China). Unless otherwise specified, all operations were carried out under aerobic circumstances. The solvents and materials applied are reagent grade and were not purified. All chemicals are commercially available and can be used without further purification, and the CO_2_ and Ar were purchased from Dehai Gas Company (Hainan, China).

#### 3.1.2. Physical Measurements

X-ray powder diffraction (PXRD) was performed on a BIGAKU DMAX2500PC diffractometer with Cu–Ka radiation (*k* = 1.54056 Å) to obtain the purity of the complement and the sample of complex **1** (Figure S2). After complex fracks with pure KBr, infrared spectral data (Figure S1) were recorded with the Nicolet 170SX infrared spectrometer (Thermo Fisher, Waltham, MA, USA) in the 4000–500 cm^−1^ scanning range. UV-vis absorption spectra were recorded on a Shimadzu 2550. MS: AB SCIEX Qtrap 5500+. Elemental analyses employ 2400 PerkinElmer analyzers to examine the percentage content of the C, H, and N elements of complex **1**.

#### 3.1.3. X-ray Crystallographic Data Collection and Refinement of the Structures

A single crystal was mounted on a Bruker SMART APEX II CCD X-ray single-crystal diffractometer (Bruker, Germany), and all data were collected at 293 K with graphite monochromated MoKa radiation (λ = 0.71073 Å) in I > 2σ(I) diffraction spots and reduced with the SAINT program, and absorption corrections were applied using the program SADABS. The structures were solved by the direct method and refined by full-matrix least-squares techniques based on *F*^2^, with all observed reflections performed with the SHELXL-2014/7 package (Software Fayre, Madrid, Spain) and Olex2 1.2 (OlexSys Ltd., Durham, UK). Table 1 gives cell parameters, spatial groups, and some conventional thermodynamic parameters and introduces the relevant crystal information in detail. Table S1 lists the selected key lengths and angles. The CCDC number for crystal is 2201814.

#### 3.1.4. Electrochemical Measurements and Electrolysis Product Analysis

To research their electrocatalytic properties, all electrochemical experiments are tested with a CHI660E electrochemical analyzer (Shanghai CHI Instruments Company, Shanghai, China), and these experiments are conducted in the single-chamber three-electrode electrolytic cell. A solution of 0.1 M ^n^Bu_4_NPF_6_ is applied as the supporting electrolyte. The pH values of the buffer are adjusted by adding 0.01 M NaOH and 0.1 M NaOH or 0.1 M HAc in sodium acetate. There is about 10 mL of buffer solution containing 0.2 mM complex **1** in the electrolytic cell. Cyclic voltammogram (CV) experiments are carried out using a glassy carbon working electrode with a diameter of 3 mm, which is seriously polished with diamond paste, ultrasonically cleaned in aqueous ethanol and deionized water, and then dried before use. The counter electrode and the reference electrode are a platinum wire electrode and a Ag/AgCl electrode, respectively. A conductive glass substrate doped with fluorine tin oxide (FTO) (1 cm × 1 cm, the effective surface area of 1.0 cm^2^) (produced by Zhuhai Kaivo Optoelectronic Corp, Zhuhai, China) is used as an operating electrode to control potential electrolysis (CPE), which is soaked with 5 wt% NaOH in ethanol solution for several hours and then washed with water, ethanol, and water in turn. Before each experiment, the solution is blown at room temperature with Ar or CO_2_ for 30 min.

In situ UV-visible spectral electrochemistry is performed by applying the constant potentials of 1.85 V and −1.99 V vs. NHE (Figure S8) to 0.1 M NaOAc and 0.1 M ^n^Bu_4_NPF_6_/DMF solution under Ar and CO_2_ atmospheres, respectively, and recorded using UV1800 UV-vis spectrophotometry (Shimadzu, Kyoto, Japan). In situ spectral electrochemistry studies employed quartz dishes with a path length of 1 cm as electrochemical batteries, including platinum mesh, platinum wire, and Ag/AgCl (saturated KCl) electrodes as working electrodes, counter electrodes, and reference electrodes, respectively. The top space gas sample (1 mL) produced by the CPE experiments is extracted using a bait-locked airtight syringe and injected into the gas chromatograph (GC, Shimadzu GC-2014, Shimadzu, Kyoto, Japan), which is equipped with a flame ionization detector (FID) containing a mechanical device to analyze carbon monoxide and equipped with a thermal conductivity detector (TCD, Shimadzu) for analysis to quantify H_2_. Detection of CO_2_ and H_2_ are carried out with ultra-high-purity Ar as carrier gas. Liquid products are analyzed by NMR (AVANCE III HD 400MHz, Brugg, Switzerland).

### 3.2. Synthesis

[Ni^II^_3_(paoH)_6_(PhPO_3_)_2_]·2ClO_4_, pyridine-2-aldoxime (0.061 g, 0.5 mmol), PhPO_3_H_2_ (0.001 g, 0.0625 mmol), Et_3_N (30 µL), and Ni(ClO_4_)_2_·6H_2_O (0.183 g, 0.5 mmol) were dissolved in 15 mL methanol (MeOH). The reaction mixture was stirred for 24 h and then filtered. The obtained green precipitate was redissolved in 10 mL acetonitrile (MeCN). Blue-green crystals suitable for X-ray diffraction were obtained by natural evaporation for about two weeks (9.07% yield, based on Ni). Calc. (Found) for C_48_H_46_C_l2_N_12_Ni_3_O_20_P_2_: C, 40.60 (40.12); H, 3.26 (3.13); N, 11.84 (11.76). Major IR wavelengths (KBr disk, cm^−1^) (Figure S1): 3159 (m), 3062 (m), 3022 (m), 2868 (m), 1620 (m), 1505 (m), 1095 (s), 1050 (s), 989 (s), 771 (w), 689 (w), 618 (w), 552 (m).

## 4. Conclusions

We have successfully constructed a trinuclear nickel complex, [Ni^II^_3_(paoH)_6_(PhPO_3_)_2_]·2ClO_4_, with two kinds of ligands, which can serve as bifunctional homogeneous molecular catalyst for water oxidation and CO_2_ reduction. Through abundant electrochemical experiments, we found that complex **1** can catalyze water oxidation with an overpotential of 560 mV under near-neutral conditions with a TOF of 12.2 s^−1^. Meanwhile, it can also catalyze the reduction of CO_2_ in DMF solution with added CH_3_COOH as a proton source, exhibiting a TOF of 7.84 s^−1^. In addition, the results of the in situ spectroelectrochemistry, the CPE tests, the rinse test, and the SEM can prove that complex **1** displays great stability for water oxidation and CO_2_ reduction. The outstanding catalytic performance of complex **1** may be due to the cooperation effect of the three nickel metal centers and, more importantly, the two unique *µ*_3_-PhPO_3_^2−^ bridges as the crucial factors for the stability of the complex, as well as due to the proton transformation during the catalytic process, together with the oxime groups tuning the electronic structure of the metal centers via π back-bonding. We believe that the exploration of the novel trinuclear Ni complex for water oxidation and CO_2_ reduction will supply valuable insights for the development of more efficient molecular catalysts.

## Figures and Tables

**Table 1:** Crystal data, data collection, and structural refinement parameters of complex **1**.

Empirical formula	C_48_H_46_Cl_2_N_12_Ni_3_O_20_P_2_
Formula weight	1419.94
Crystal system	monoclinic
Space group	*P2/n*
a [Å]	12.9435(7)
b [Å]	19.4313(11)
c [Å]	13.9832(8)
*α* [°]	90
*β* [°]	103.797(5)
*γ* [°]	90
*V* [Å^3^]	3415.4(3)
*Z*	2
*ρ_calcd_* [g m^−3^]	1.381
*μ* [mm^−1^]	2.753 mm^−1^
Reflections collected	13,086
F(000)	1452
*R_int_*	0.05
T [K]	293(2)K
Final *R* indices	*R*_1_ = 0.0525
[*I* > 2sigma(I)]	*wR*_1_ = 0.1723
*R* indices (all data)	*R*_2_ = 0.0677
	*wR*_2_ = 0.1957
Gof	0.85
[a] R_1_ = ∑||F_o_| − |F_c_||/∑|F_o_|, _w_R_2_ = [∑_w_(F_o_^2−^ − F_c_^2^)^2^]/[∑_w_(F_o_^2^)^2^]^1/2^	

## Data Availability

Data are contained within the article and Supplementary Materials.

## Supplementary Materials

The following supporting information can be downloaded at: https://www.mdpi.com/article/10.3390/molecules29030578/s1. XPS of the structures, electrochemical measurements and electrolysis product analysis, SEM images, PXRD, infrared spectra, CV curves, in situ UV-vis spectroelectrochemistry, etc., can be found in the Supplementary Materials.
