# Supplementary material for: Electrochemical Water Oxidation and CO2 Reduction with a Nickel Molecular Catalyst
Source: Molecules. 2024 Jan 24;29(3):578. doi: 10.3390/molecules29030578 (PMC10856054; doi:10.3390/molecules29030578)
Supplement: Supplementary file 1 [file molecules-29-00578-s001.zip › molecules-2817784-supplementary.pdf]

SUPPORTING INFORMATION:

Electrochemical Water Oxidation and CO_2 Reduction by a Nickel Molecular Catalyst

Hengxin Jian[1], Mengyu Lu[1], Haowen Zheng[1], Shengrui Yan[1] and Mei Wang*[1]

Contents **Page No**

Figure S1. Infrared spectra of complex 1 2

Figure S2. Figure S2. XPS spectra of complex 1. .... Error! Bookmark not defined.

Figure S3. In NaOAc solution, cyclic voltammetry curves of 0.2 mM complex 1 and two kinds of ligands under Ar atmosphere at pH = 7.45. 4

Figure S4. Cyclic voltammetry curves of 0.2 mM complex 1 and double ligands under CO_2 atmosphere in DMF solution including 0.1 M $^nBu_4NPF_6$. (at scanning rate = 100 mVs^{-1}) 5

Figure S6. Cyclic voltammograms of 0.2 mM complex 1 (red line) and 10 M NaOH (blank line) in 0.1 M aqueous sodium acetate solution. (At scanning rate of 100 mV s^{-1}, using glassy carbon electrode as a working electrode) 7

Figure S7. Before and after CPE, the surface morphology of the FTO glass electrode was observed. (a) Before electrolysis (b) Water oxidation, after electrolysis (c) CO_2 reduction, after electrolysis. 8

Figure S8. Continuous 10 scan cycles of (a) water oxidation of 0.2 mM complex 1 in 0.1 M NaOAc buffer solution at pH 7.45 and after (b) CO_2 reduction of 0.2 mM complex 1 in 0.1 M $^nBu_4NPF_6$ DMF solution. (scan rate = 100 mV/s) 9

Figure S9. The in-situ UV-Vis spectroelectrochemistry of complex 1 during 4000s CPE (a) water oxidation and (b) CO_2 reduction. 10

Figure S10. Schematic diagram of In-situ UV-visible spectral electrochemistry. 11

Table S1. The main bond length of complex 1. 12

Table S2. Comparison of nickel-based water oxidation catalysts with different TOF values in recent years. 13

Table S3. The products and FE of CO_2 reduction. 13

Table S4. Comparison of nickel-based CO_2 reduction catalysts with different TOF values in recent years. 13

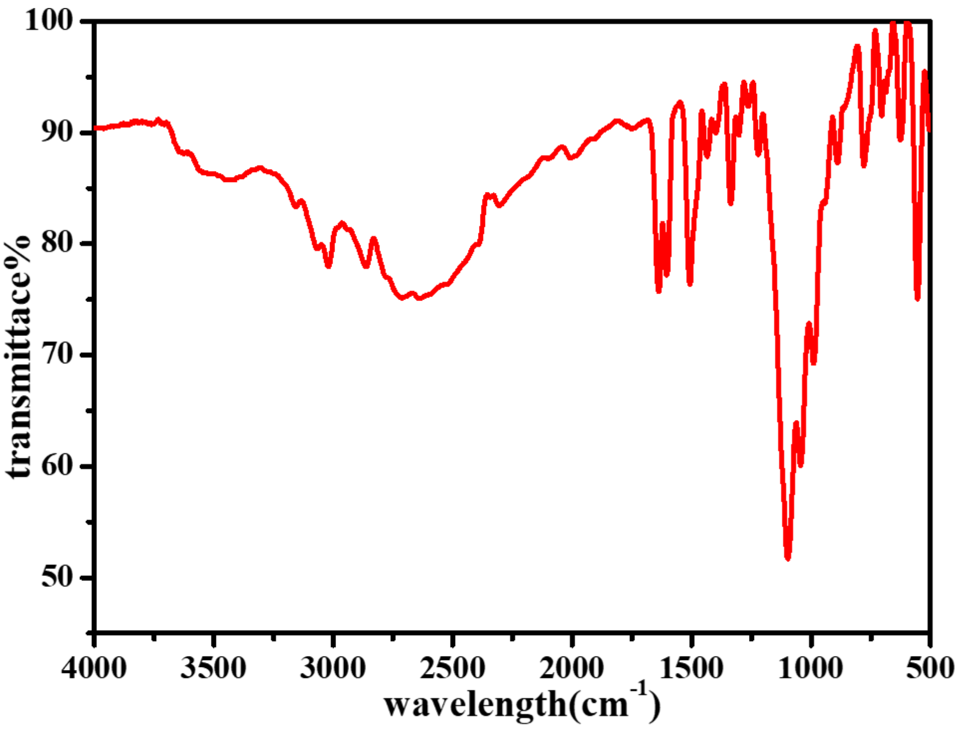

Figure S1. Infrared spectra of complex **1**

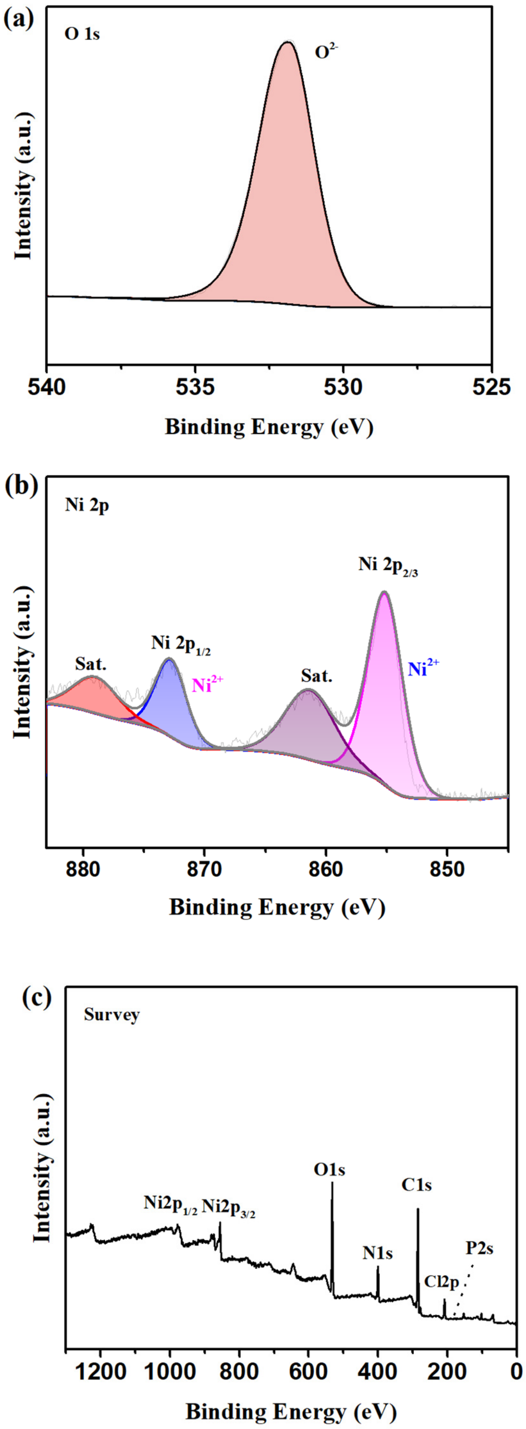

Figure S2. XPS spectra of complex **1.**

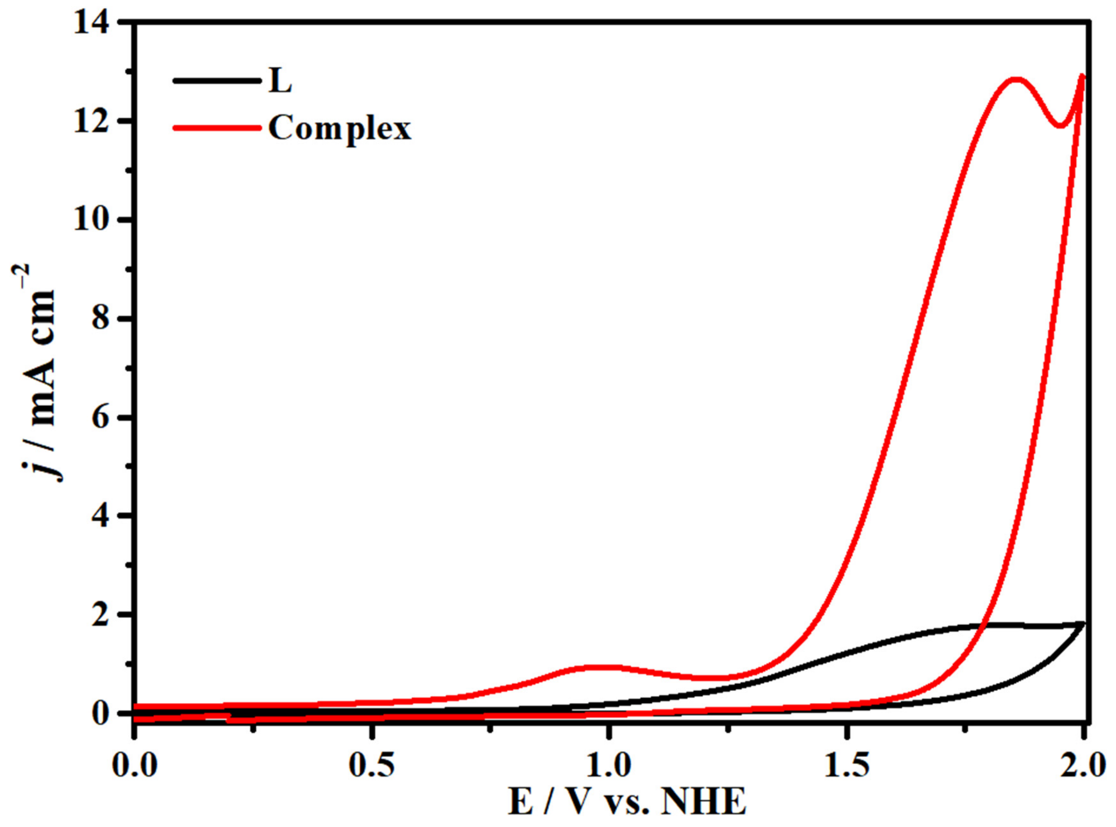

Figure S3. In NaOAc solution, cyclic voltammetry curves of 0.2 mM complex **1** and two kinds of ligands under Ar atmosphere at pH = 7.45.

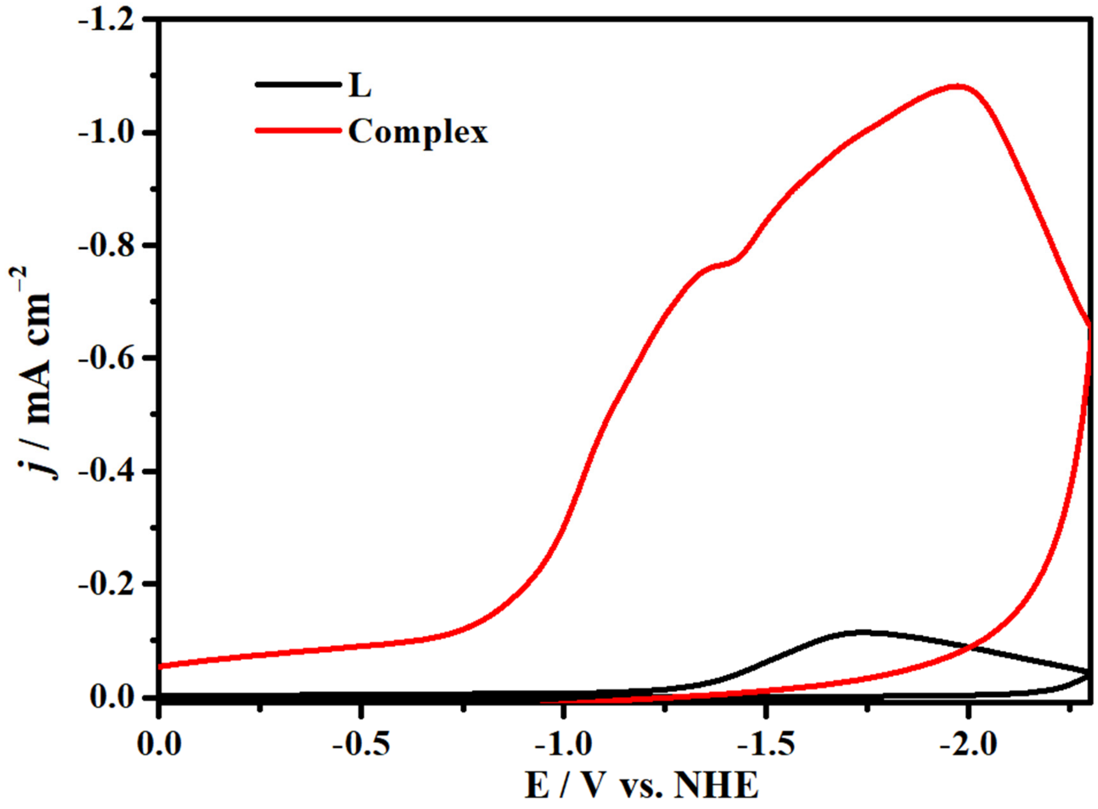

Figure S4. Cyclic voltammetry curves of 0.2 mM complex **1** and double ligands under CO_2 atmosphere in DMF solution including 0.1 M $^{n}Bu_4NPF_6$. (at scanning rate = 100 mVs^{-1})

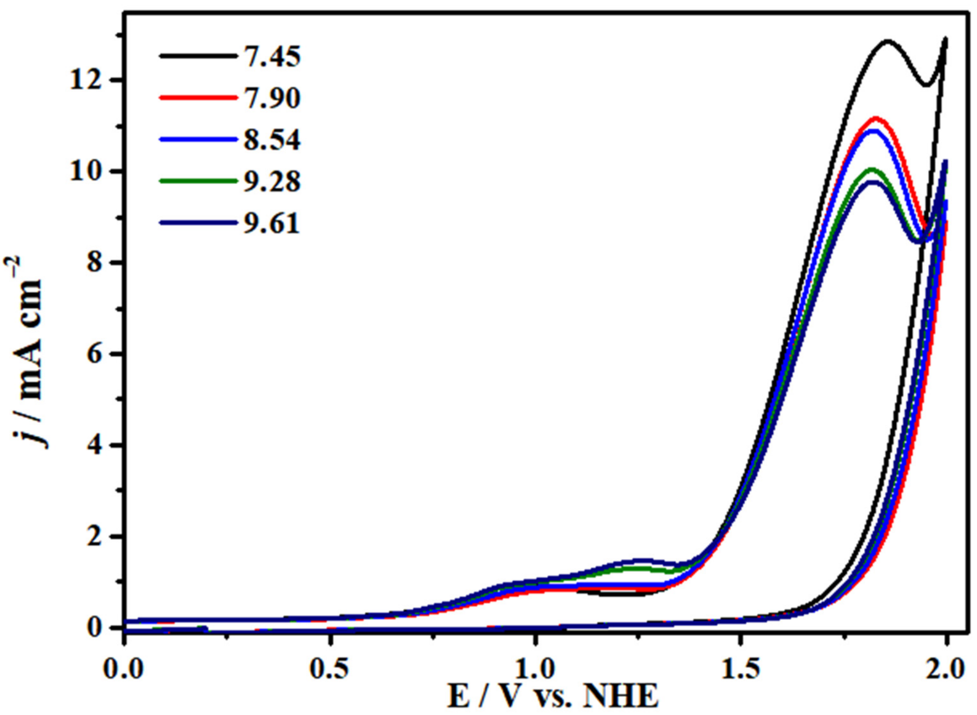

Figure S5. Cyclic voltammograms of 0.1 M NaOH solution added to 0.2 mM complex 1 in 0.1 M aqueous sodium acetate solution at different pH.

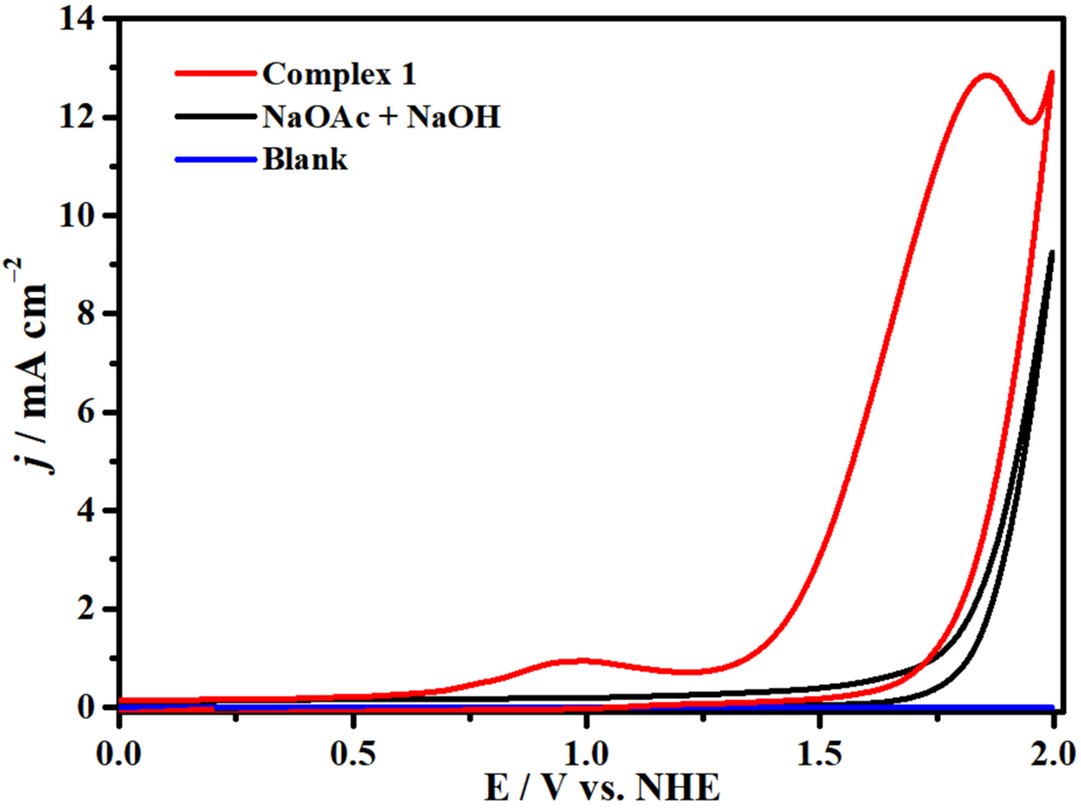

Figure S6. Cyclic voltammograms of 0.2 mM complex **1** (red line) and 10 M NaOH (blank line) in 0.1 M aqueous sodium acetate solution. (At scanning rate of 100 mV s^{-1}, using glassy carbon electrode as a working electrode)

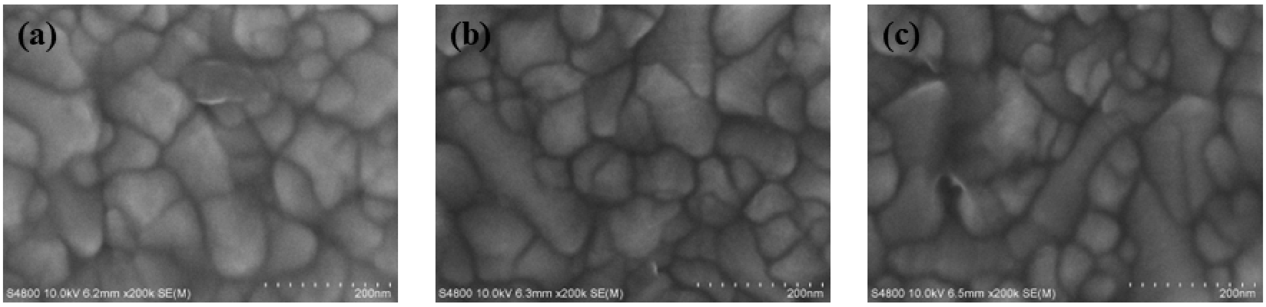

Figure S7. Before and after CPE, the surface morphology of the FTO glass electrode was observed. (a) Before electrolysis (b) Water oxidation, after electrolysis (c) CO_2 reduction, after electrolysis.

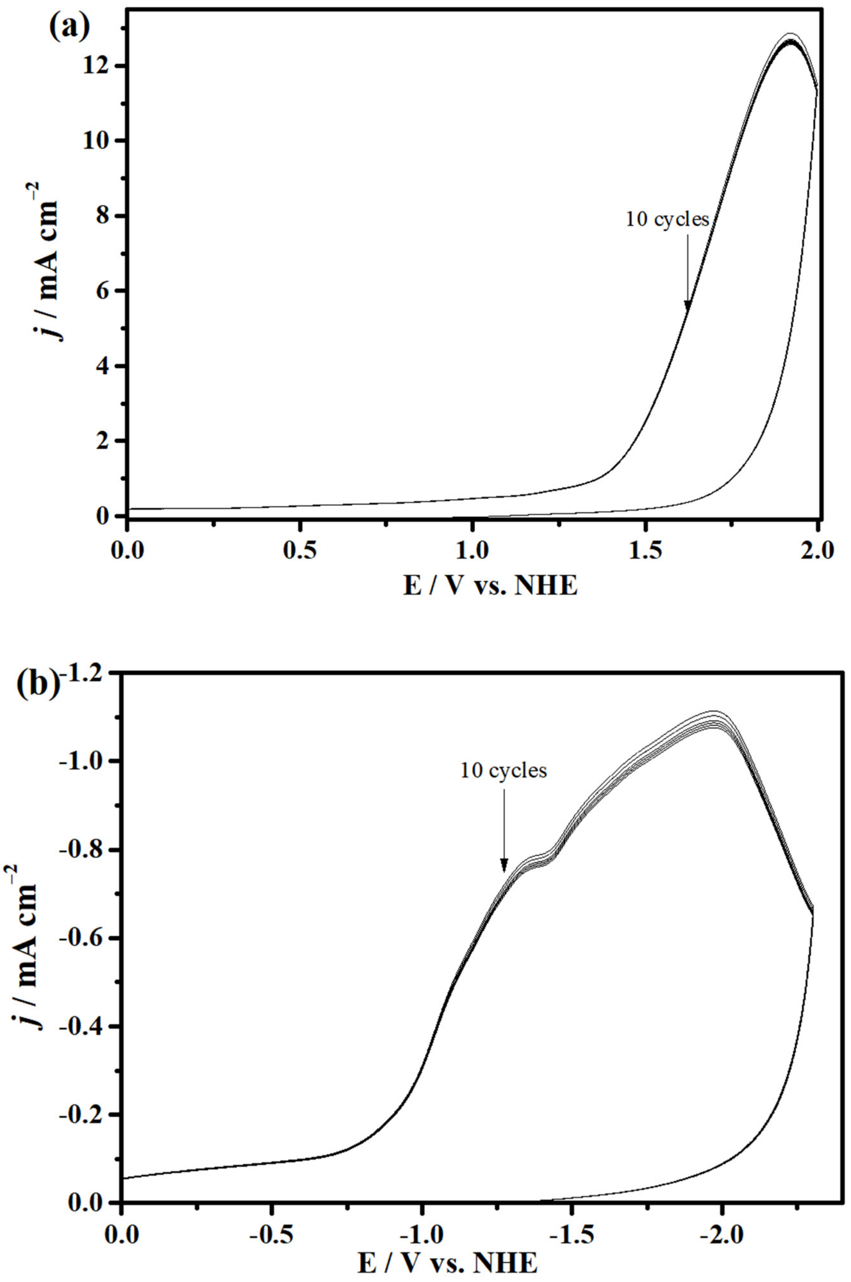

Figure S8. Continuous 10 scan cycles of (a) water oxidation of 0.2 mM complex **1** in 0.1 M NaOAc buffer solution at pH 7.45 and after (b) CO_2 reduction of 0.2 mM complex **1** in 0.1 M $^{n}Bu_4NPF_6$ DMF solution. (scan rate = 100 mV/s)

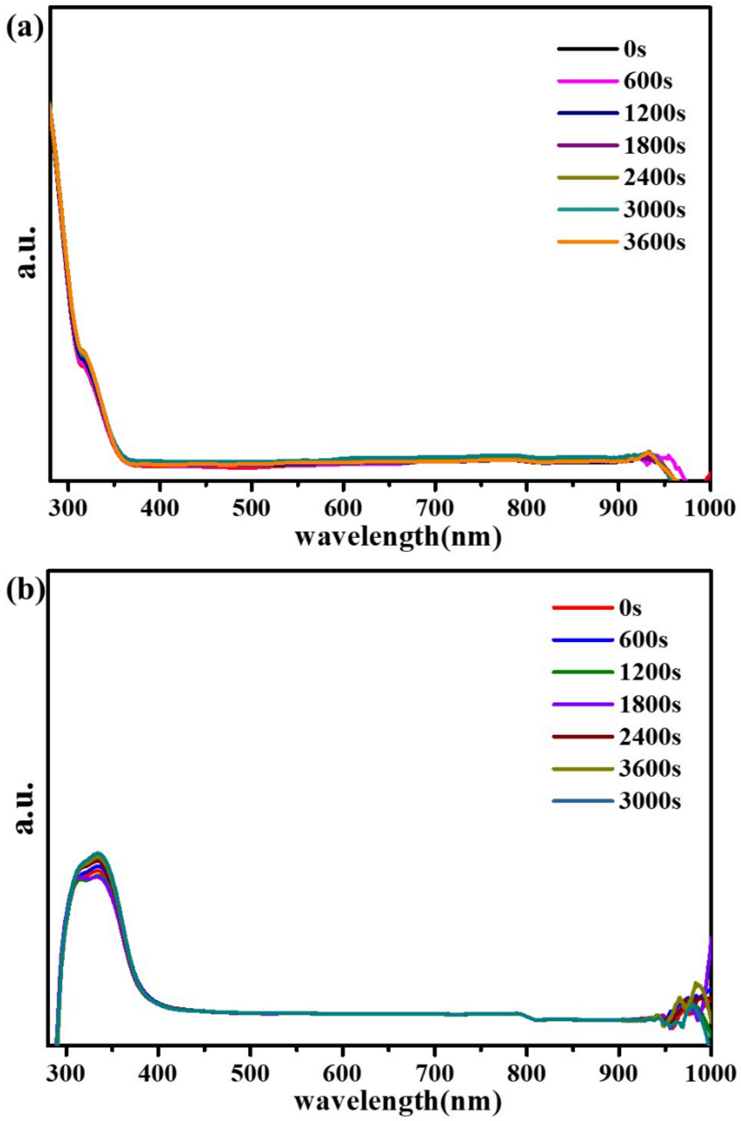

Figure S9. The in-situ UV-Vis spectroelectrochemistry of complex **1** during 4000s CPE (a) water oxidation and (b) CO_2 reduction.

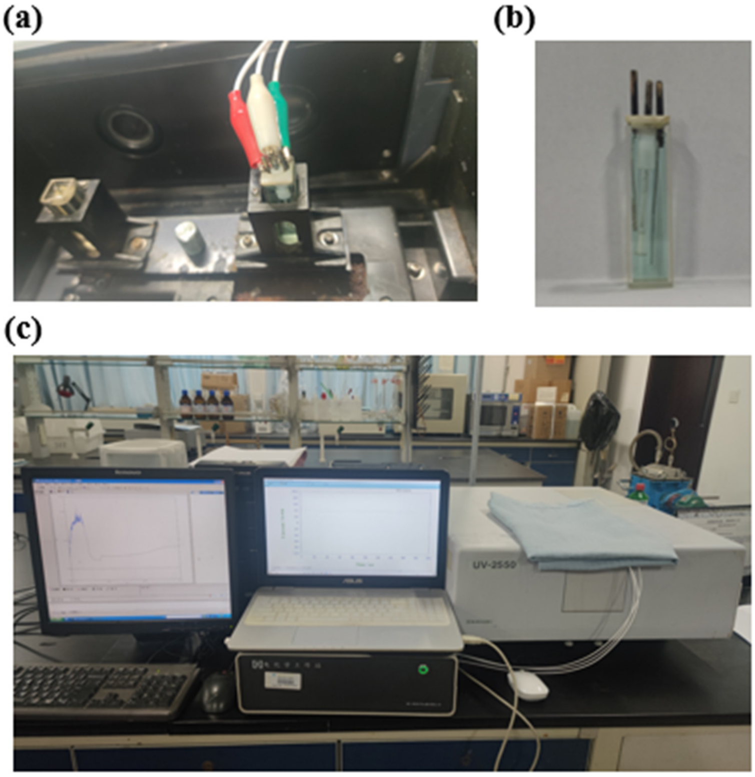

Figure S10. Schematic diagram of In-situ UV-visible spectral electrochemistry.

Table S1. The main bond length of complex **1.**

Bond	Length (Å)	Bond	Length (Å)
Ni1-O2	2.055(2)	Ni1-N6	2.096(3)
Ni1-O2	2.055(2)	Ni1-N6	2.096(3)
Ni1-N5	2.094(3)	Ni2-O1	2.060(2)
Ni1-N5	2.094(3)	Ni2-N2	2.076(3)
Ni2-O3	2.078(2)	Ni2-N1	2.109(3)
Ni2-N4	2.083(3)	Ni2-N3	2.116(3)

The main bond angles of complex **1.**

Bond	Angle (°)	Bond	Angle (°)
O2-Ni1-N5	172.01(11)	N5-Ni1-N6	95.22(13)
O2-Ni1-N5	88.99(12)	N6-Ni1-N6	169.71(17)
O2-Ni1-N5	88.99(12)	O1-Ni2-N2	90.81(11)
O2-Ni1-N5	172.01(11)	O1-Ni2-O3	97.98(9)
N5-Ni1-N5	87.9(2)	N2-Ni2-O3	95.26(11)
O2-Ni1-N6	91.27(10)	O1-Ni2-N4	95.99(11)
O2-Ni1-N6	95.68(10)	N2-Ni2-N4	170.08(12)
N5-Ni1-N6	95.22(13)	O3-Ni2-N4	90.95(11)
N5-Ni1-N6	77.28(12)	O1-Ni2-N1	86.88(11)
O2-Ni1-N6	95.68(10)	N2-Ni2-N1	77.22(13)
O2-Ni1-N6	91.27(10)	O3-Ni2-N1	171.17(11)
N5-Ni1-N6	77.28(12)	N4-Ni2-N1	95.90(13)
O1-Ni2-N3	171.32(11)	C5-N1-Ni2	113.3(2)
N2-Ni2-N3	95.37(13)	C6-N2-Ni2	116.7(3)
O3-Ni2-N3	87.53(11)	O4-N2-Ni2	129.0(2)
N4-Ni2-N3	77.14(13)	C7-N3-Ni2	128.7(3)
N1-Ni2-N3	88.55(13)	C11-N3-Ni2	113.5(3)
O5-N4-Ni2	129.3(2)	O6-N6-Ni1	130.0(2)
C13-N5-Ni1	129.3(3)	P1-O1-Ni2	131.46(14)
C17-N5-Ni1	113.2(3)	P1-O2-Ni1	133.23(14)
C18-N6-Ni1	115.6(3)	P1-O3-Ni2	129.35(14)

Table S2. Comparison of nickel-based water oxidation catalysts with different TOF values in recent years.

catalyst	η(mV)	TOF(s^{-1})	FE	
$(Me_4N)_2[Ni^{II}L^i]$		0.4		Ref. 1
α-$Ni(OH)_2$	350	0.036		Ref. 2
$[Ni^{II}(tpen)](ClO_4)_2\cdot 0.5CH_3COCH_3$	440	0.04	89%	Ref. 3
$Na_2[Ni^{II}$ (TAML)]	680	0.32	93%	Ref. 4
$[Ni(TMC)(CH_3CN)](NO_3)_2$	590	9.95		Ref. 5
$[NiL(H_2O)_2](ClO4)_2$	753		70%	Ref. 6
**$[Ni^{II}_3(paoH)_6(PhPO_3)_2]\cdot 2ClO_4$**	**560**	**12.2**	**95%**	**This work**

Table S3. The products and FE of CO_2 reduction.

With or without CH_3COOH	Time (s)	CO yield (PPM)	H_2 yield (PPM)	FE_{CO}	FE_{H_2}
With CH_3COOH	1500	51.4	208.0	7.62	57.34
	2000	156.2	828.63	17.37	92.11
	3000	75.9	1272.8	5.63	94.32
	4000	38.8	1743.2	2.16	96.89
Without CH_3COOH	1500	80.74	762.0	7.81	73.71
	2000	168.9	1304.8	12.26	94.66
	3000	143.5	1994.6	6.94	96.47
	4000	119.3	2727.4	4.33	98.93

Table S4. Comparison of nickel-based CO_2 reduction catalysts with different TOF values in recent years.

catalyst	TOF(s^{-1})	
$[bn_4cyclenNiCl]Cl$	8	Ref. 7
Carbazolide-bis(NHC) Ni^{II}	1	Ref.8
$[Ni^{II}(Me_3NTB)(CH_3CN)_2](BF_4)_2$	22.1	Ref. 9
**$[Ni^{II}_3(paoH)_6(PhPO_3)_2]\cdot 2ClO_4$**	**7.84**	**This work**

References:

1. Lin, J.; Kang, P.; Liang, X.; Ma, B.; Ding, Y. Homogeneous electrocatalytic water oxidation catalyzed by a mononuclear nickel complex. Electrochim. Acta **2017**, 258, 353-359.
2. Hussain, N.; Yang, W.; Dou, J.; Chen, Y.; Qian, Y.; Xu, L. Ultrathin mesoporous F-doped α-$Ni(OH)_2$ nanosheets as an efficient electrode material for water splitting and supercapacitors. J. Mater. Chem. A **2019**, 7, 9656-9664.
3. Li, Q.-J.; Ren, Y.-J.; Xie, Q.; Wu, M.; Feng, H.-X.; Zheng, L.-M.; Zhang, H.-X.; Long, J.-Q.; Wang, T.-S. Nickel (II) tetrapyridyl complexes as electrocatalysts and precatalysts for water oxidation. Appl Organomet Chem **2020**, 34, e5813.
4. Lee, H.; Wu, X.; Sun, L. Copper-based homogeneous and heterogeneous catalysts for electrochemical water oxidation. Nanoscale **2020**, 12, 4187-4218.
5. Zhang, L.-H.; Yu, F.; Shi, Y.; Li, F.; Li, H. Base-enhanced electrochemical water oxidation by a nickel complex in neutral aqueous solution. ChemComm **2019**, 55, 6122-6125.
6. Luo, G.-Y.; Huang, H.-H.; Wang, J.-W.; Lu, T.-B. Further Investigation of a Nickel-Based Homogeneous Water Oxidation Catalyst with Two cis Labile Sites. ChemSusChem **2016**, 9, 485-491.
7. Md. Ahsan, H.; Breedlove, B. K.; Cosquer, G.; Yamashita, M. Enhancement of electrocatalytic abilities toward CO_2 reduction by tethering redox-active metal complexes to the active site. Dalton Trans. **2021**, 50, 13368-13373.
8. Huang, H.-H.; Zhang, J.-H.; Dai, M.; Liu, L.; Ye, Z.; Liu, J.; Zhong, D.-C.; Wang, J.-W.; Zhao, C.; Ke, Z. Dual electronic effects achieving a high-performance Ni(II) pincer catalyst for CO_2 photoreduction in a noble-metal-free system.*Proc. Natl. Acad. Sci. U.S.A.* **2022**, 119, e2119267119
9. Wang, J.-W.; Huang, H.-H.; Sun, J.-K.; Zhong, D.-C.; Lu, T.-B. Syngas Production with a Highly-Robust Nickel(II) Homogeneous Electrocatalyst in a Water-Containing System. ACS Catal. **2018**, 8, 7612-7620.
